# Supplementary material for: Brain tumor classification in VIT-B/16 based on relative position encoding and residual MLP
Source: PLoS One. 2024 Jul 2;19(7):e0298102. doi: 10.1371/journal.pone.0298102 (PMC11218980; doi:10.1371/journal.pone.0298102)
Supplement: Attachment — Submitted filename: Response to Reviewers.pdf [file pone.0298102.s003.pdf]

We sincerely thank the editor and all reviewers for spending time on our draft and providing thoughtful comments. We will consider all the comments in the revised version. The point-to-point response to all the comments are listed as follows.

Q1. Reviewer #1: In the related work only CNN, U-Net and simple neural networks are explored, not any related work of Vision Transformers quoted.

Answer: Thank you for the suggestion. In response to valuable suggestion from the reviewers, following an analysis of the fundamental aspects of the Vision Transformer model, we have incorporated references to some relevant works in the field.

Q2. Reviewer #1: A typo mistake in the paper is in images where Homomorphic Filtering abbreviation is HF while in images in section 3, it is abbreviated as HE.

Answer: Thank you for the suggestion. I have corrected this spelling error and reviewed the remaining sections of my paper.

Q3. Reviewer #1: Only classification of Brain Tumor was done, as the Vision Transformers are used i.e. without CNN therefore no pixel level segmentation done.

Answer: Thank you for the suggestion. I have carefully considered your comment regarding the classification of brain tumors based on Vision Transformers and the absence of pixel-level segmentation in my study. I would like to provide further context to address your concerns and explain the rationale behind my approach. The primary objective of my research was to investigate the efficacy of Vision Transformers for brain tumor classification tasks. My study aimed to explore the potential of ViT in accurately classifying different types of brain tumors based on high-level features extracted from medical images. Given the inherent complexity of pixel-level segmentation and its resource-intensive nature, I made a deliberate decision to focus on classification using ViT. While I acknowledge the importance of pixel-level segmentation in the field of medical image analysis, the availability of suitable datasets for this specific task played a crucial role in shaping the scope of my study. My dataset was not conducive to pixel-level segmentation due to its limitations in terms of scale and annotation complexity.

Q4. Reviewer #1: In the evaluation matric only accuracy is used, for generalization performance it is necessary to evaluate models by precision, F1 score, Jaccard similarity index etc.

Answer: Thank you for the suggestion. We have now included precision, recall rate, F1 score, and other relevant evaluation metrics in the experiments section of the paper. These metrics provide a more holistic assessment of our model's performance, particularly in the context of brain tumor classification. To aid in the interpretation of our results, we have included visualizations of precision-recall curves and other relevant plots in the " The training process of the experiments" section. These visualizations offer a more intuitive representation of our model's performance.

Q5. Reviewer #2: Due to unbalance dataset data, your result are imprecise. you should

add some precision, sensitivity, recall and MCC to improve the accuracy of your measurements.

Answer: Thank you for the suggestion. We have now included precision, recall rate, F1 score, and other relevant evaluation metrics in the experiments section of the paper. These metrics provide a more holistic assessment of our model's performance, particularly in the context of brain tumor classification. To aid in the interpretation of our results, we have included visualizations of precision-recall curves and other relevant plots in the " The training process of the experiments" section. These visualizations offer a more intuitive representation of our model's performance.

Q6. Reviewer #2: The author should add the precision recall curve and feature space visualization on the base of PCA.

Answer: Thank you for the suggestion. In response to your recommendation to add PCA-based feature space visualization, we carefully considered the best approach to provide meaningful insights into our model's performance and feature representations. Upon further evaluation, we decided to include precision-recall curves and visualization results of transformer encoder blocks instead of PCA-based visualization for the following reasons: Our proposed approach leverages transformer encoder blocks, which play a central role in our model architecture. Visualizing the feature space within these blocks provides a direct and contextually relevant representation of how our model processes input images. Precision-recall curves are well-established and widely used in evaluating classification models, especially in medical applications where the balance between precision and recall is crucial. Including these curves allows for a more direct interpretation of our model's performance in the context of brain tumor classification. The visualization results of transformer encoder blocks offer insights into the hierarchical feature representations learned by our model. These visualizations contribute to a comprehensive understanding of how our model captures and processes information, which aligns with the objectives of our research. While we understand your initial suggestion to incorporate PCA-based feature space visualization, we believe that the inclusion of precision-recall curves and transformer encoder block visualizations provides a more relevant and informative assessment of our model's performance and feature representations in the context of brain tumor classification. We hope that this explanation clarifies our rationale for the chosen visualization methods and addresses your concerns.

Q7. Reviewer #2: This paper claims significant improvement in classification of accuracy compared to VIT-B/16.However, it would be more meaningful to compare the proposed method with other state-of-the-art brain tumor classification algorithms, including CNN-base models and other advanced techniques, and includes novelty.

Answer: Thank you for the suggestion. We have expanded the comparative analysis in our paper to include EfficientNet, in addition to the previously mentioned CNN-based models (AlexNet, VGGNet, GoogLeNet, ResNet) and the lightweight neural network MobileNet. This enhancement allows for a more robust evaluation of our proposed method's performance in the context of brain tumor classification. Furthermore, in the related work section of the paper, we have included citations of recent and innovative brain tumor classification methods. We have also highlighted the novelty and advancement of our approach.

Q8. Reviewer #2: Vision Transformer model. Not only used for classification method but can also use for segmentation methods. You should include more points in this paper for future and also mention innovation in this paper.

Answer: Thank you for the suggestion. I have carefully considered your comment regarding the classification of brain tumors based on Vision Transformers and the absence of pixel-level segmentation in my study. I would like to provide further context to address your concerns and explain the rationale behind my approach. The primary objective of my research was to investigate the efficacy of Vision Transformers for brain tumor classification tasks. My study aimed to explore the potential of ViT in accurately classifying different types of brain tumors based on high-level features extracted from medical images. Given the inherent complexity of pixel-level segmentation and its resource-intensive nature, I made a deliberate decision to focus on classification using ViT. While I acknowledge the importance of pixel-level segmentation in the field of medical image analysis, the availability of suitable datasets for this specific task played a crucial role in shaping the scope of my study. My dataset was not conducive to pixel-level segmentation due to its limitations in terms of scale and annotation complexity.

Q9. Reviewer #2: The introduction and literature section can be strengthened by discussing the recent deep CNNs work on various medical applications like breast cancer:

• Khan, Saddam Hussain, Javed Iqbal, Syed Agha Hassnain, Muhammad Owais, Samih M. Mostafa, Myriam Hadjouni, and Amena Mahmoud. "Covid-19 detection and analysis from lung ct images using novel channel boosted cnns." Expert Systems with Applications 229 (2023): 120477.

• Khan, Saddam Hussain, Asifullah Khan, Yeon Soo Lee, Mehdi Hassan, and Woong Kyo Jeong. "Segmentation of shoulder muscle MRI using a new region and edge based deep auto-encoder." Multimedia Tools and Applications 82, no. 10 (2023): 14963-14984.

• Rauf, Zunaira, Anabia Sohail, Saddam Hussain Khan, Asifullah Khan, Jeonghwan Gwak, and Muhammad Maqbool. "Attention-guided multi-scale deep object detection framework for lymphocyte analysis in IHC histological images." Microscopy 72, no. 1 (2023): 27-42.

• Khan, Saddam Hussain. "Malaria Parasitic Detection using a New Deep Boosted and Ensemble Learning Framework." arXiv preprint arXiv:2212.02477 (2022).

• Khan, Saddam Hussain, Najmus Saher Shah, Rabia Nuzhat, Abdul Majid, Hani Alquhayz, and Asifullah Khan. "Malaria parasite classification framework using a novel channel squeezed and boosted CNN." Microscopy 71, no. 5 (2022): 271-282.

Answer: Thank you for the suggestion. As per your suggestion, I have restructured the 'Related Work' section and actively cited the reference materials you recommended to discuss the role of convolutional neural networks in medical applications.

Q10. Reviewer #3: Abstract section is well written but , need to be more be concise and precise

Firstly, an abstract should summarize the major aspects of the entire paper:

- The overall purpose of the study
- Major findings as a result of your analysis
- Dataset details
- Mention the validation technique
- Clinical feasibility and impact not discussed
- A brief summary of your interpretations and conclusions.

Answer: Thank you for the suggestion. We have revised the abstract to provide a more concise and precise summary of our research. We have clarified the overarching purpose of our study, emphasizing its relevance to brain tumor classification. In other sections, I have made the necessary modifications as well, in accordance with the requirements you outlined.

Q11. Reviewer #3: The Introduction section is well-written, but it would benefit from more conciseness and improved flow and rhythm. Limited discussion on the specific challenges in brain tumor diagnosis and data statistics and sources. Moreover, the contribution section requires enhancements in effectively conveying crucial information about the Vision Transformer model, such as its novelty, rationale, and impact. Furthermore, discussion on the limitations of Vision Transformers. Additionally, the research contributions need to be clearly described as they currently lack clarity and are limited in scope in terms of ViT.

Answer: Thank you for the suggestion. We have reorganized and rewritten the Introduction section to address the following key points, We have carefully revised the content to ensure conciseness while maintaining a logical flow and rhythm. This means that the Introduction provides a clear and engaging overview of our research. We have included a more detailed discussion of the specific challenges in brain tumor diagnosis, encompassing aspects such as complex structure, texture, size, location, and appearance. And acknowledging the limitations of available brain tumor datasets, particularly in terms of size and availability. We have enriched the Introduction with crucial information about the Vision Transformer model, emphasizing its novelty, rationale, and potential impact in the field of brain tumor classification.

Q12. Reviewer #3: Research Gap: Insufficient analysis of drawbacks in previous brain tumor classification approaches, lack of evidence for limitations in traditional CNNs in medical image tasks and limited discussion on the limitations of the Vision Transformer.

Answer: Thank you for the suggestion. We have conducted a more comprehensive analysis of the drawbacks of previous brain tumor classification approaches. This expanded discussion not only identifies the limitations of existing methods but also highlights the need for our proposed approach. The related work section has been rewritten to provide a more in-depth and insightful overview of prior research in the field of brain tumor classification. This revision ensures that readers better understand the existing landscape and the context of our work. We have incorporated a dedicated discussion on the limitations of the Vision Transformer

model in the Introduction and Conclusion sections. This addition allows us to provide a well-rounded assessment of our proposed approach and the model's constraints.

Q13. Reviewer #3: The system configuration and software application used to perform the work needs to be written properly in Section "Experiment and Analysis".

Answer: Thank you for the suggestion. We have correctly documented the system configuration and software applications used for conducting the work in the 'Experiments and Analysis' section

Q14. Reviewer #3: Explanation of the relative position encoding method, Iinsufficient justification for enhancing the MLP with a residual network, and limited insight into the generalization capability improvement of the model.

Answer: Thank you for the suggestion. We have provided a more detailed explanation of the relative position encoding method used in our research. This enhancement ensures that readers have a clearer understanding of this critical component of our approach. We also have included additional justification for the enhancement of the MLP with a residual network. By expanding on the rationale and benefits of this modification, we aim to provide a more comprehensive understanding of our design choices.

Q15. Reviewer #3: The paper lacks ablation experimental analysis to verify the effectiveness of various designs implemented in their proposed model. For instance, there is no analysis regarding the contribution of data augmentation to the model's performance. Additionally, to provide a more comprehensive performance analysis, it would be beneficial to include a comparison of the proposed model's performance with other state-of-the-art methods.

Answer: Thank you for the suggestion. In Section 3.4, Table 6's second row indicates the accuracy improvement of the original VIT model after incorporating data augmentation techniques. In the subsequent paragraph, an analysis is provided regarding the enhancement in model performance attributed to the utilization of image augmentation. We have expanded the comparative analysis in our paper to include EfficientNet, in addition to the previously mentioned CNN-based models (AlexNet, VGGNet, GoogLeNet, ResNet) and the lightweight neural network MobileNet. This enhancement allows for a more robust evaluation of our proposed method's performance in the context of brain tumor classification. Furthermore, in the related work section of the paper, we have included citations of recent and innovative brain tumor classification methods. We have also highlighted the novelty and advancement of our approach.

Q16. Reviewer #3: The performance analysis of the proposed model has been solely compared using accuracy. However, since the employed dataset is unbalanced in nature, accuracy alone is not sufficient for a comprehensive performance comparison. Incorporating additional performance measures such as Recall, Precision, F-score, AUC, and ROC would provide more robust evaluation metrics, especially when dealing with unbalanced datasets.

Answer: Thank you for the suggestion. We have now included precision, recall rate, F1 score, and other relevant evaluation metrics in the experiments section of the paper. These metrics provide a more holistic assessment of our model's performance, particularly in the context of brain tumor classification. To aid in the interpretation of our results, we have included visualizations of precision-recall curves and other relevant plots in the " The training process of the experiments" section. These visualizations offer a more intuitive representation of our model's performance.

Q17. Reviewer #3: To enhance the quality of your work, it is crucial to address any limitations present within it. Moreover, providing an expansion on future directions can offer valuable insights for further research and development.

Answer: Thank you for the suggestion. We have conducted an analysis of the limitations and deficiencies associated with the Vision Transformer in our study. This allowed us to identify areas for improvement, which we have addressed and documented in the paper. We have expanded the concluding section of our paper to provide insights into future directions for research and development in the field of brain tumor classification. These suggestions are aimed at guiding researchers and practitioners toward valuable areas for further exploration and innovation.

Q18. Reviewer #3: The conclusion section should also include a discussion of the limitations and potential pitfalls of deep learning and ViT models in medical applications.

Answer: Thank you for the suggestion. In the conclusion section, we have a discussion on the limitations and potential pitfalls of deep learning and Vision Transformer models in medical applications. This discussion offers a critical perspective on the challenges and considerations inherent in utilizing such models for medical tasks. By incorporating these insights, we aim to provide a more balanced and holistic view of the implications of deep learning and ViT models in the medical domain.

Q19. Reviewer #3: Please, cite the fallowing paper to strengthened related works to brain tumor and other medical application:

• Zahoor, M. M., & Khan, S. H. (2022). Brain tumor MRI Classification using a Novel Deep Residual and Regional CNN. arXiv preprint arXiv:2211.16571.

• Khan, S. H., Sohail, A., Zafar, M. M., & Khan, A. (2021). Coronavirus disease analysis using chest X-ray images and a novel deep convolutional neural network. Photodiagnosis and Photodynamic Therapy, 35, 102473.

• Zahoor, M.M.; Qureshi, S.A.; Bibi, S.; Khan, S.H.; Khan, A.; Ghafoor, U.; Bhutta, M.R. A New Deep Hybrid Boosted and Ensemble Learning-Based Brain Tumor Analysis Using MRI. Sensors 2022, 22, 2726. https://doi.org/10.3390/s22072726

• Khan, S. H., Sohail, A., Khan, A., Hassan, M., Lee, Y. S., Alam, J., ... & Zubair, S. (2021). COVID-19 detection in chest X-ray images using deep boosted hybrid learning. Computers in Biology and Medicine, 137, 104816.

• Khan, Asifullah, Saddam Hussain Khan, Mahrukh Saif, Asiya Batool, Anabia

Sohail, and Muhammad Waleed Khan. "A Survey of Deep Learning Techniques for the Analysis of COVID-19 and their usability for Detecting Omicron." Journal of Experimental & Theoretical Artificial Intelligence (2023): 1-43.

Answer: Thank you for the suggestion. We have rewritten the 'Related Work' section and incorporated citations to several reference papers that you recommended. These additions enhance the depth and relevance of our literature review, providing a more comprehensive understanding of the research landscape.
