# Supplementary material for: Brain tumor classification in VIT-B/16 based on relative position encoding and residual MLP
Source: PLoS One. 2024 Jul 2;19(7):e0298102. doi: 10.1371/journal.pone.0298102 (PMC11218980; doi:10.1371/journal.pone.0298102)
Supplement: Attachment — Submitted filename: Response to Reviewers.pdf [file pone.0298102.s004.pdf]

We sincerely thank the editor and all reviewers for spending time on our draft and providing thoughtful comments. We will consider all the comments in the revised version. The point-to-point response to all the comments are listed as follows:

Q1. Reviewer #4: Reference must be corrected.

Answer: Thank you for the suggestion. In response to your and the editors' guidance, I have meticulously reviewed and updated the references, aiming to enhance the overall precision and correctness of the citation and bibliography. The modifications to the references are as follows:

1. Bray F, Ferlay J, Soerjomataram I, et al. Global cancer statistics 2018: GLOBOCAN estimates of incidence and mortality worldwide for 36 cancers in 185 countries[J]. CA: a cancer journal for clinicians, 2018, 68(6): 394-424.
2. Liu D, Zhang H, Zhao M, et al. Brain Tumor Segmentation Based on Dilated Convolution Refine Networks. 2018 IEEE 16th International Conference on Software Engineering Research, Management and Applications (SERA).IEEE, 2018: 113-120. https://doi.org/10.1109/sera.2018.8477213
3. Khan, Saddam Hussain. Malaria Parasitic Detection using a New Deep Boosted and Ensemble Learning Framework. Converg. Inf. Ind. Telecommun. Broadcast. data Process. 1981-1996, vol. 26, no. 1, pp. 125–150, Dec. 2022.
4. Khan A, Rauf Z, Khan A R, et al. A Recent Survey of Vision Transformers for Medical Image Segmentation[J]. arXiv preprint ArXiv abs/2312.00634 (2023): n. pag.
5. Srivastava, Nitish et al. Dropout: a simple way to prevent neural networks from overfitting. J. Mach. Learn. Res. 15 (2014): 1929-1958.
6. Bai J, Yuan L, Xia S T, et al. Improving vision transformers by revisiting high-frequency components[C]. European Conference on Computer Vision(ECCV). Cham: Springer Nature Switzerland, 2022: 1-18.
7. M. Buckland and F. Gey. The relationship between recall and precision[J]. Journal of the American Society for Information Science, 1994, 45(1): 12-19.
8. M. Sokolova, N. Japkowicz, and S. Szpakowicz. Beyond accuracy, F-score and ROC: a family of discriminant measures for performance evaluation[C]. Australasian joint conference on artificial intelligence. Berlin, Heidelberg: Springer Berlin Heidelberg, 2006: 1015-1021.
9. S. Liu and W. Deng. Very deep convolutional neural network based image classification using small training sample size. 2015 3rd IAPR Asian Conference on Pattern Recognition (ACPR), Kuala Lumpur, Malaysia, 2015, pp. 730-734, doi: 10.1109/ACPR.2015.7486599.
10. Szegedy C, Liu W, Jia Y, et al. Going deeper with convolutions. Proceedings of the IEEE Conference on Computer Vision and Pattern Recognition (CVPR), 2015: 1-9

Q2. Reviewer #4: Reference must be corrected.

Answer: Thank you for the suggestion. Regarding your revised opinion on "Statistical test like confidential interval", I conducted experiments on the confidence level related to model accuracy in the article and made corresponding analysis.

Q3. Reviewer #4: Please, clarify the novelty.

Answer: Thank you for the suggestion. In response to the query regarding the novelty of our work, I have made revisions to the abstract and introduction sections of the

manuscript to explicitly highlight the unique contributions and innovations of our study.

Q4. Reviewer #4: PR curve of all the model must be provided.

Answer: Thank you for the suggestion. In response to this valuable suggestion, we have made the necessary enhancements, the PR curves for all the models are now included in the article. The additional figures offer a comprehensive view of the performance of each model in terms of precision and recall. We believe that these additions significantly contribute to the overall clarity and transparency of our findings.
