# Supplementary material for: Brain tumor classification in VIT-B/16 based on relative position encoding and residual MLP
Source: PLoS One. 2024 Jul 2;19(7):e0298102. doi: 10.1371/journal.pone.0298102 (PMC11218980; doi:10.1371/journal.pone.0298102)
Supplement: Attachment — Submitted filename: Response to Reviewers.pdf [file pone.0298102.s005.pdf]

*We sincerely thank the editor and all reviewers for spending time on our draft and providing thoughtful comments. We will consider all the comments in the revised version. The point-to-point response to all the comments are listed as follows.*

**Q1. Reviewer #1: the authors addressed most of the reviewer comments and the article is at the level to accept. However, some future direction must required**

Answer: Thank you for the suggestion. In response to your suggestion regarding the inclusion of future research directions, we have added a detailed section in the conclusion of our manuscript. This section outlines several key areas for future research based on the content of our article and the current status of research in the field.

**Q2. Reviewer #2: It is suggested that authors need to compare their classification results with up-to-date models, not just compare with traditional convolutional neural networks.**

Answer: Thank you for the suggestion. We would like to clarify that our comparative experiments include not only traditional CNNs but also relatively new and advanced CNN architectures. Specifically, we have incorporated MobileNet and EfficientNet, which are recognized for their state-of-the-art performance in various image classification tasks. One of our primary research goals is to compare the superiority of the Vision Transformer (ViT) with CNNs in the context of brain tumor classification.

**Q3. Reviewer #2: Each image patch has been projected into a vector of dimension 768 after Flatten in Figure 1, so no image patch should be drawn after Flatten. A similar situation also appears in Figure 3, where the input to the Transformer block is some vectors, so the input to the self-attention should be a vector instead of a convolutional feature map.**

Answer: Thank you for the suggestion. We acknowledge your point that each image patch is projected into a vector of dimension 768 after the flattening operation in the figures. You are correct that, after this operation, the image patches should no longer be drawn as patches but rather represented as vectors. We have revised the figures. We have made these corrections in the revised manuscript to ensure that the figures accurately represent the data flow and transformations within our model. These updates will provide clearer visual guidance and prevent any potential misunderstandings regarding the operations involved in our methodology.

**Q4. Reviewer #2: The original version of the Vision Transformer (ViT) already has two residual connections in each Transformer block (one for the multi-head self-attention part and the other for the MLP part), and the authors have plotted the residual connections for the multi-head self-attention part in Figure 1. Perhaps the authors missed the residual connection of the MLP part in Figure 1. In addition, the authors need to explain the advantages of the residual connection designed in this paper and the original residual connection in ViT.**

Answer: Thank you for the suggestion. We have revised Figure 1 to ensure that the figure accurately represents the architecture of the original ViT model. This update should clarify

any misunderstandings regarding the residual connections in our model. Regarding the innovation in our work, we have designed a novel residual structure specifically for the MLP part of the ViT model. This innovation aims to enhance the performance and stability of the model. Our residual structure is different from the one in the original ViT model, as it introduces an additional pathway that allows the MLP part to better capture and propagate features. To clarify, our focus was on demonstrating the effectiveness of this new residual structure within the MLP of the ViT model rather than comparing it with the existing residual connections in the original model. We believe that our proposed residual structure provides significant advantages in terms of enhancing feature representation and improving classification performance, as demonstrated in our experimental results.

**Q5. Reviewer #2: Why did the authors not split the validation set when splitting the dataset?**

Answer: Thank you for the suggestion. We split our dataset into training and test sets in a ratio of 4:1. The primary reason for not including a separate validation set is the relatively limited size of our brain tumor dataset. Allocating data to a validation set in addition to the test set would further reduce the number of samples available for training, potentially affecting the model's ability to learn effectively. To ensure the robustness and reliability of our model, we employed cross-validation techniques during the training phase. That allows us to validate the model's performance iteratively and ensure that it generalizes well without the need for a separate validation set. This approach maximizes the use of available data while providing a reliable estimate of model performance. By using cross-validation, we are able to fine-tune the model and perform hyperparameter optimization effectively. The final evaluation is then performed on the test set, which remains unseen during the training and validation phases, providing an unbiased assessment of the model's performance. We believe that this approach ensures the best possible use of our dataset, maintaining a balance between training data sufficiency and model validation.

**Q6. Reviewer #2: According to the test set curve in Figure 7, the author seems to use the test set as a validation set. The test set should not actually be used for training. Only after the model is trained, the model can be tested on the test set and the test results can be obtained.**

Answer: Thank you for the suggestion. I apologize for any confusion caused by the depiction in the figure. To clarify, the test set was not used for training the model. The model was trained exclusively on the training set. The intention behind plotting the accuracy trend on the test set after each epoch was to provide a clear visualization of how the model's performance generalizes over time. The accuracy trend shown in the figure reflects the model's performance on the test set at the end of each epoch, which helps in understanding the model's generalization capabilities and potential overfitting. This approach allows us to monitor the model's performance without influencing the training process with test data.

We understand the importance of keeping the test set completely separate from the training and validation processes. Therefore, we can assure you that the test set was only used for final evaluation after the model was fully trained. We hope this explanation addresses your concerns and clarifies our methodology. Thank you again for your constructive comments.

We look forward to your feedback on the revised manuscript.
