# Supplementary material for: Brain tumor classification in VIT-B/16 based on relative position encoding and residual MLP
Source: PLoS One. 2024 Jul 2;19(7):e0298102. doi: 10.1371/journal.pone.0298102 (PMC11218980; doi:10.1371/journal.pone.0298102)
Supplement: Attachment — Submitted filename: paper_review[1].docx [file pone.0298102.s001.docx]

Brain Tumor Classification in VIT-B/16 based on Relative Position Encoding and

Residual MLP

--Manuscript Draft—

Feedback :

The proposed paper made valuable contributions to brain tumor classification but there are some areas where improvement may be necessary.

1. Due to unbalance dataset data, your result are imprecise. you should add some precision, sensitivity, recall and MCC to improve the accuracy of your measurements.
2. The author should add the precision recall curve and feature space visualization on the base of PCA.
3. This paper claims significant improvement in classification of accuracy compared to VIT-B/16.However, it would be more meaningful to compare the proposed method with other state-of-the-art brain tumor classification algorithms, including CNN-base models and other advanced techniques, and includes novelty.
4. Vision Transformer model. Not only used for classification method but can also use for segmentation methods. You should include more points in this paper for future and also mention innovation in this paper.
5. The introduction and literature section can be strengthened by discussing the recent deep CNNs work on various medical applications like breast cancer:

- Khan, Saddam Hussain, Javed Iqbal, Syed Agha Hassnain, Muhammad Owais, Samih M. Mostafa, Myriam Hadjouni, and Amena Mahmoud. "Covid-19 detection and analysis from lung ct images using novel channel boosted cnns." Expert Systems with Applications 229 (2023): 120477.
- Khan, Saddam Hussain, Asifullah Khan, Yeon Soo Lee, Mehdi Hassan, and Woong Kyo Jeong. "Segmentation of shoulder muscle MRI using a new region and edge based deep auto-encoder." Multimedia Tools and Applications 82, no. 10 (2023): 14963-14984.
- Rauf, Zunaira, Anabia Sohail, Saddam Hussain Khan, Asifullah Khan, Jeonghwan Gwak, and Muhammad Maqbool. "Attention-guided multi-scale deep object detection framework for lymphocyte analysis in IHC histological images." Microscopy 72, no. 1 (2023): 27-42.
- Khan, Saddam Hussain. "Malaria Parasitic Detection using a New Deep Boosted and Ensemble Learning Framework." arXiv preprint arXiv:2212.02477 (2022).
- Khan, Saddam Hussain, Najmus Saher Shah, Rabia Nuzhat, Abdul Majid, Hani Alquhayz, and Asifullah Khan. "Malaria parasite classification framework using a novel channel squeezed and boosted CNN." Microscopy 71, no. 5 (2022): 271-282.
