# Supplementary material for: Brain tumor classification in VIT-B/16 based on relative position encoding and residual MLP
Source: PLoS One. 2024 Jul 2;19(7):e0298102. doi: 10.1371/journal.pone.0298102 (PMC11218980; doi:10.1371/journal.pone.0298102)
Supplement: Attachment — Submitted filename: Review_PLOS_Brain_Tumor_Classification_in_VIT-B16_based_on_Relative_Position_Encoding_and_Residual_MLP[1].docx [file pone.0298102.s002.docx]

This work introduces an enhanced Vision Transformer model as an improved algorithm for human brain tumor classification. However, the paper is challenging to follow coherently, and the innovation and contribution of the proposed model are not clearly highlighted. The model appears to be a simple residual network structure, which is already well-established in the field of deep learning. Moreover, the evaluation of the proposed model is solely compared to the standard CNN, serving as a baseline but not representing the cutting-edge technology. Although the authors present corresponding experimental results, the overall experiments lack specificity, and the experimental design is not rigorous enough. Below are some specific comments that may provide more clarity and constructive feedback:

1. Abstract section is well written but , need to be more be concise and precise

Firstly, an abstract should summarize the major aspects of the entire paper:

- The overall purpose of the study
- Major findings as a result of your analysis
- Dataset details
- Mention the validation technique
- Clinical feasibility and impact not discussed
- A brief summary of your interpretations and conclusions.

2. The Introduction section is well-written, but it would benefit from more conciseness and improved flow and rhythm. Limited discussion on the specific challenges in brain tumor diagnosis and data statistics and sources. Moreover, the contribution section requires enhancements in effectively conveying crucial information about the Vision Transformer model, such as its novelty, rationale, and impact. Furthermore, discussion on the limitations of Vision Transformers. Additionally, the research contributions need to be clearly described as they currently lack clarity and are limited in scope in terms of ViT.
3. Research Gap: Insufficient analysis of drawbacks in previous brain tumor classification approaches, lack of evidence for limitations in traditional CNNs in medical image tasks and limited discussion on the limitations of the Vision Transformer.
4. The system configuration and software application used to perform the work needs to be written properly in Section “Experiment and Analysis”.
5. Explanation of the relative position encoding method, Iinsufficient justification for enhancing the MLP with a residual network, and limited insight into the generalization capability improvement of the model.
6. The paper lacks ablation experimental analysis to verify the effectiveness of various designs implemented in their proposed model. For instance, there is no analysis regarding the contribution of data augmentation to the model's performance. Additionally, to provide a more comprehensive performance analysis, it would be beneficial to include a comparison of the proposed model's performance with other state-of-the-art methods.
7. The performance analysis of the proposed model has been solely compared using accuracy. However, since the employed dataset is unbalanced in nature, accuracy alone is not sufficient for a comprehensive performance comparison. Incorporating additional performance measures such as Recall, Precision, F-score, AUC, and ROC would provide more robust evaluation metrics, especially when dealing with unbalanced datasets.
8. To enhance the quality of your work, it is crucial to address any limitations present within it. Moreover, providing an expansion on future directions can offer valuable insights for further research and development.
9. The conclusion section should also include a discussion of the limitations and potential pitfalls of deep learning and ViT models in medical applications.
10. Please, cite the fallowing paper to strengthened related works to brain tumor and other medical application:

- Zahoor, M. M., & Khan, S. H. (2022). Brain tumor MRI Classification using a Novel Deep Residual and Regional CNN. arXiv preprint arXiv:2211.16571.
- Khan, S. H., Sohail, A., Zafar, M. M., & Khan, A. (2021). Coronavirus disease analysis using chest X-ray images and a novel deep convolutional neural network. Photodiagnosis and Photodynamic Therapy, 35, 102473.
- Zahoor, M.M.; Qureshi, S.A.; Bibi, S.; Khan, S.H.; Khan, A.; Ghafoor, U.; Bhutta, M.R. A New Deep Hybrid Boosted and Ensemble Learning-Based Brain Tumor Analysis Using MRI. Sensors 2022, 22, 2726. https://doi.org/10.3390/s22072726
- Khan, S. H., Sohail, A., Khan, A., Hassan, M., Lee, Y. S., Alam, J., ... & Zubair, S. (2021). COVID-19 detection in chest X-ray images using deep boosted hybrid learning. Computers in Biology and Medicine, 137, 104816.
- Khan, Asifullah, Saddam Hussain Khan, Mahrukh Saif, Asiya Batool, Anabia Sohail, and Muhammad Waleed Khan. "A Survey of Deep Learning Techniques for the Analysis of COVID-19 and their usability for Detecting Omicron." Journal of Experimental & Theoretical Artificial Intelligence (2023): 1-43.
